# Slight Variations in the Sequence Downstream of the Polyadenylation Signal Significantly Increase Transgene Expression in HEK293T and CHO Cells

**DOI:** 10.3390/ijms232415485

**Published:** 2022-12-07

**Authors:** Evgeniya S. Omelina, Anna E. Letiagina, Lidiya V. Boldyreva, Anna A. Ogienko, Yuliya A. Galimova, Lyubov A. Yarinich, Alexey V. Pindyurin, Evgeniya N. Andreyeva

**Affiliations:** 1 Department of Regulation of Genetic Processes, Institute of Molecular and Cellular Biology, SB RAS, 630090 Novosibirsk, Russia; 2 Laboratory of Biotechnology, Novosibirsk State Agrarian University, 630039 Novosibirsk, Russia; 3 Laboratory of Experimental Models of Cognitive and Emotional Disorders, Scientific-Research Institute of Neurosciences and Medicine, 630117 Novosibirsk, Russia

**Keywords:** gene expression regulation, transcription termination, massively parallel reporter assay, MPRA, barcode, next-generation sequencing, NGS

## Abstract

Compared to transcription initiation, much less is known about transcription termination. In particular, large-scale mutagenesis studies have, so far, primarily concentrated on promoter and enhancer, but not terminator sequences. Here, we used a massively parallel reporter assay (MPRA) to systematically analyze the influence of short (8 bp) sequence variants (mutations) located downstream of the polyadenylation signal (PAS) on the steady-state mRNA level of the upstream gene, employing an *eGFP* reporter and human HEK293T cells as a model system. In total, we evaluated 227,755 mutations located at different overlapping positions within +17..+56 bp downstream of the PAS for their ability to regulate the reporter gene expression. We found that the positions +17..+44 bp downstream of the PAS are more essential for gene upregulation than those located more distal to the PAS, and that the mutation sequences ensuring high levels of *eGFP* mRNA expression are extremely T-rich. Next, we validated the positive effect of a couple of mutations identified in the MPRA screening on the *eGFP* and luciferase protein expression. The most promising mutation increased the expression of the reporter proteins 13-fold and sevenfold on average in HEK293T and CHO cells, respectively. Overall, these findings might be useful for further improving the efficiency of production of therapeutic products, e.g., recombinant antibodies.

## 1. Introduction

In eukaryotes, the termination of the transcription process can substantially influence the level of gene expression via various mechanisms [1,2,3]. Moreover, gene expression regulation was shown to be primarily controlled by transcription termination in those systems, where background transcription prevails [4,5]. Lesions in the transcription termination were found to be associated with oncological, immunological, neurological, and other diseases [6,7,8,9,10,11,12]. Transcription termination of protein-coding genes relies on the assembly of the functional cleavage and polyadenylation (CPA) complex at the 3′ end of the newly synthesized transcripts [13,14]. This process is tightly coordinated with pre-mRNA synthesis, capping, and splicing [15,16,17,18,19]. The mammalian CPA complex encompasses more than 80 subunits that are grouped into several functional subcomplexes [20,21,22]. Three subcomplexes (cleavage and polyadenylation specificity factor (CPSF), cleavage stimulation factor (CstF), and cleavage factor Im (CF Im)) bind directly to pre-mRNA to form a core complex, which is then accomplished by CF IIm, Symplekin, and poly(A) polymerase (PAP), resulting in a functional CPA complex [20,21]. Assembly of this complex is initiated at a hexamer sequence referred to as the polyadenylation signal (PAS) (AAUAAA and its multiple close variations), during the transcription elongation, and requires the presence of the C-terminal domain of the RNA polymerase II (RNAP II) large subunit that undergoes a set of covalent modifications [23,24,25]. CPSF and CstF subcomplexes recognize the PAS and the downstream sequence element (DSE) in pre-mRNAs, respectively [22,26,27,28]. CPSF is additionally subdivided into two modules by their function: mPSF (mammalian polyadenylation specificity factor) that binds the PAS and recruits PAP, thus catalyzing the polyadenylation process, and mCF that catalyzes the pre-mRNA cleavage reaction [14,29,30]. The CF Im subcomplex is known to bind the actively elongating RNAP II together with CPSF and CstF [31]. CF Im recognizes the UGUA sequence (and its variations), which can be located either upstream or downstream of the mature transcript cleavage site [31,32,33,34], and it stabilizes binding of the CPSF to the primary transcript [35]. The CF Im factor can also bind two UGUA motifs due to the capability of dimerization of its CFI25 subunit, resulting in a loop at the 3′ end of pre-mRNA [36]. This feature, as well as the correlation of increased CF Im levels with alternative polyadenylation (APA), suggests its significant role in the PAS selection during APA [34,37,38]. Several additional subcomplexes play a role in the CPA complex assembly and can influence the efficiency of pre-mRNA processing. The cleavage factor IIm (CF IIm) subcomplex remains one of the less studied factors in the mammalian CPA complex. It is highly homologous to proteins involved in transcription termination in yeast [39,40]. The yeast homolog of one of the CF IIm subunits interacts directly with the C-terminal domain of RNAP II [41,42] and other CPA components, and its knockdown causes impaired transcription termination [43,44]. For another CF IIm subunit homolog, the interaction and functional interrelation with CPSF and CF Im were shown [45,46]. Symplekin protein was also shown to interact directly with several subunits of the CPA complex [20,47]. However, its exact role has not been determined so far. Possibly, Symplekin stabilizes the whole CPA complex, being a multi-binding protein. There is also evidence that Symplekin can act as a regulator of the nuclease activity of the CPSF-73 subunit of mCF by binding additional regulatory factors [48,49,50]. PAP was found in eukaryotes in four variants (PAP, Neo-PAP, Star-PAP, and TPAP) [51,52]. PAP is known to directly interact with subunits of CPSF and CF Im [53], but many studies have indicated that PAP is not stably associated with the CPA complex [54,55], and this may reflect a highly dynamic organization of the core 3′ processing complex. An additional number of proteins that are involved in the PAS recognition and can modulate the activity of the CPA complex were also found [56,57,58,59]. At the same time, it was shown that only CPSF and PAP are necessary and sufficient for polyadenylation of transcripts in vitro [21,60,61]. Thus, the CPA complex is a huge multicomponent machine, which assembly and activity depend on various factors.

The mechanisms and key factors of transcription termination remain much less understood than the processes of transcription initiation and elongation. Two main hypothetical schemes describing the mechanism of transcription termination in mammals have been discussed during the last 30 years. The *allosteric* model suggests that the CPA complex assembly results in a RNAP II conformation change that leads to the loss of elongation factors and, thus, further synthesis of the RNA chain stops [62,63]. This model is confirmed by the fact that RNAP II ceases to bind elongation factors much earlier than it dissociates from the synthesized pre-RNA and DNA template [64,65]. The *torpedo* model is based on the assumption that elongating RNAP II meets the exonuclease activity of the CPA complex, resulting in cleavage of pre-mRNA from the 3′ end. The interaction of the CPA complex with RNAP II leads to the dissociation of both complexes [66,67]. The *torpedo* model better explains the relationship between transcript cleavage and termination and is supported by the experimentally shown “sliding” of RNA synthesis in the absence of the 5′–3′ exoribonuclease 2 (XRN2) activity [68]. Recently, the combined *allosteric/torpedo* mechanism was proposed, in which termination factor-dependent slowing down of RNAP II within the termination regions facilitates its capture by XRN2 and subsequent 3’ -> 5’ degradation of the 3’ pre-mRNA end [69].

Instability and variations in the CPA complex assembly, as well as the stochastic nature of the cleavage process, result in APA through the selection of a particular one of multiple PASs [9,56,70,71]. Therefore, the length and composition of mRNA 3′ untranslated regions (UTRs) vary, resulting in different sets of targets for RNA-binding proteins and miRNAs, which finally define the mRNA transport, lifespan, and translation efficiency [71,72,73]. In addition, the length of the poly(A) tail is also crucial for the same aspects of mRNA biology [70,74,75].

The particular variant of the CPA complex assembly and the PAS choice depend also on the nucleotide composition of the 3′ region of the immature transcript [76]. Consensus nucleotide motifs that induce and improve the CPA complex assembly are still not well defined. Known motifs are located both upstream and downstream of the pre-mRNA cleavage site, and the latter are, therefore, not included in the mature mRNAs [77,78,79]. Despite numerous attempts, no consensus has yet been found to be associated with the transcript cleavage site during transcription termination. The most well-known, but not the only, transcript cleavage site was found immediately after the CA dinucleotide [77,80,81]. In addition, the DSE is often found ~40 bp downstream of the transcript cleavage site, although about 20% of human mRNAs do not contain such a motif [20,76,77]. To date, it is believed that the transcription termination process is based only on RNA–protein interactions. It was shown that minimal functional PAS requires only a strong DSE and an A-rich upstream sequence in human cells [82]. Mature mRNAs were also found to have AU-rich elements (AREs) in their 3′ UTR regions, although these motifs can also be located near the 5′ end of the transcript. Binding a range of RNA-binding proteins (RBP), the ARE element is known to induce mRNA degradation [83,84]. For instance, early response genes that are sensitive to a wide range of external signals, including oncogenes and cytokines, have a relatively short lifespan of mRNAs due to AREs [85]. However, other RBPs, e.g., HuR, by binding to ARE elements, support mRNA stability by preventing endonuclease access [86]. It is extremely curious that none of the abovementioned motifs is found in all mammalian pre-mRNA molecules. The PAS is also present in only about 70% of mature human mRNAs [3,79,87]. Thus, a detailed functional study of the mechanism(s) of transcription termination with regard to the regulatory capacity of nucleotide motifs at the gene 3’ end is still relevant for understanding the regulation of expression of protein-coding genes in mammals.

Previously, in transient transfection assays, we showed that a single cytosine deletion at the position +32 bp downstream of the single PAS (deltaC) causes a ~2-fold increase in *eGFP* reporter gene expression in mouse embryonic stem cells (mESCs), cultured mouse 3T3 cells, and human embryonic kidney (HEK293T) cells [88,89], indicating its involvement in a molecular mechanism that is likely to be conserved in mammals. This deltaC appears to be involved in regulating the choice of the cleavage site, as the most proximal site (located 14 bp downstream of the PAS) becomes prevalent upon this single-nucleotide deletion. In addition, the replacement of several 16 bp fragments downstream of the PAS with artificial sequences of the same length showed that regions at +25..+40 and +33..+48 bp relative to the PAS are most sensitive to nucleotide variations and revealed particular sequences (at +25..+40 bp relative to the PAS) that increase *eGFP* expression up to fourfold [89].

In the present work, we performed a functional massively parallel reporter assay (MPRA) to assess the capability of 8 bp long mutations located within the +17..+56 bp region downstream of the PAS to control the mRNA level of the upstream *eGFP* reporter in HEK293T cells. As a result, we found that mutations significantly increasing *eGFP* expression are extremely T-rich. Next, we validated the positive effect of several mutations identified in the MPRA screening on *eGFP* expression at the protein level in HEK293T and Chinese hamster ovary (CHO) cells. Lastly, for a couple of chosen mutations, we demonstrated that they also increase the expression level of the luciferase reporter both in HEK293T and CHO cells.

## 2. Results

MPRAs are based on the usage of highly diverse plasmid libraries, which contain two key fragments, a sequence under study (a region of interest, ROI) and a barcode (BC). BCs and ROIs are usually short sequences located within and outside of the transcription unit, respectively. Thus, BCs can be used to quantify the effects of different ROI sequences that are absent in mature transcripts on the abundance of the latter in transfected cells [90,91].

**Figure 1:**
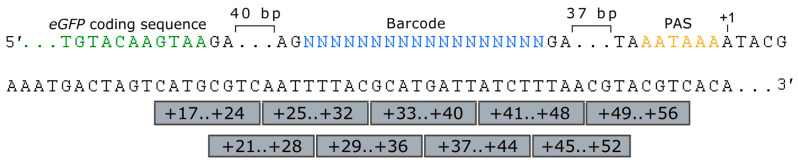
Structure of MPRA libraries (using the example of the sequence of the wildtype reference construct) used to assess an influence of sequences located downstream of the PAS on the *eGFP* reporter gene expression. PAS—polyadenylation signal (AATAAA). ROIs are shown as gray boxes. The nucleotide sequence of the 3′ end of the *eGFP* coding sequence is shown in green. Here, “40 bp” and “37 bp” designate spacers, whose nucleotide sequences are not shown for simplicity reason.

In this study, we aimed to systematically analyze the influence of nucleotide content at different positions downstream of the PAS on the steady-state mRNA levels of the upstream reporter gene in human HEK293T cells. To do that, we constructed nine MPRA plasmid libraries in which BCs and ROIs are located in the 3′ UTR of the *eGFP* reporter gene and downstream of the PAS, respectively (Figure 1). ROIs were 8 bp sequences located at positions within +17 and +56 bp relative to the PAS. Each ROI was mutagenized using random oligonucleotide primers. BCs were also random sequences but of a substantially longer length (18 bp). For the normalization purpose, an equimolar pool of two reference constructs with the original “wildtype” sequence of the transcription terminator tagged by specific 20 bp BCs was spiked at a ratio of 1/100 in each MPRA plasmid library. HEK293T cells transfected with the MPRA libraries were harvested for the assessment of the BC abundance in the *eGFP* transcripts 48 h after transfection.

**Figure 2:**
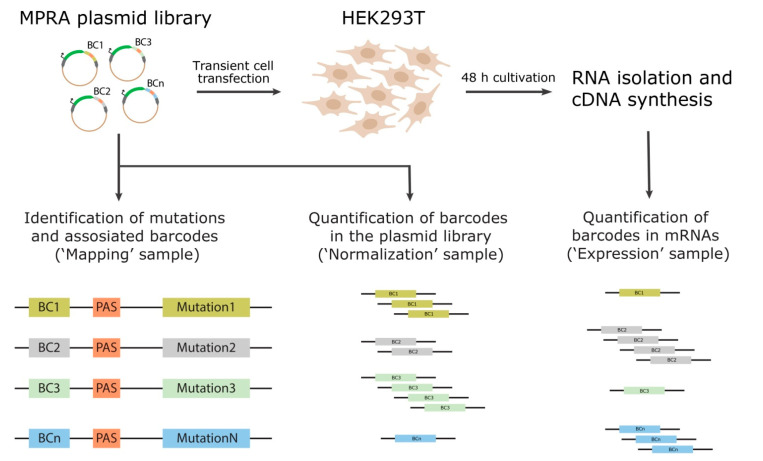
Schematic showing the experimental steps involved in the preparation of MPRA samples for subsequent NGS analysis. BC—barcode; PAS—polyadenylation signal.

Since we used MPRA libraries with *a priori* unknown sequences of BCs and ROIs, it was necessary to prepare the “mapping” samples to identify unique BC–ROI-associated sequences (Figure 2). This was achieved by performing two-round conventional PCR amplification of the BC–ROI regions using the plasmid libraries as a template [92]. MPRA “expression” and “normalization” samples were prepared by PCR amplification of the BC sequences using cDNA obtained from the transfected cells and the plasmid library DNA as a template, respectively (Figure 2). The ratio of each BC abundance in the expression and normalization samples allows judging the influence of the corresponding ROI sequence variant (hereafter mutation) on the reporter gene expression. The “mapping”, “normalization”, and “expression” samples were subjected to NGS and, on average, ~1.5 million 151 nt single-end reads were obtained for each sample replicate.

**Figure 3:**
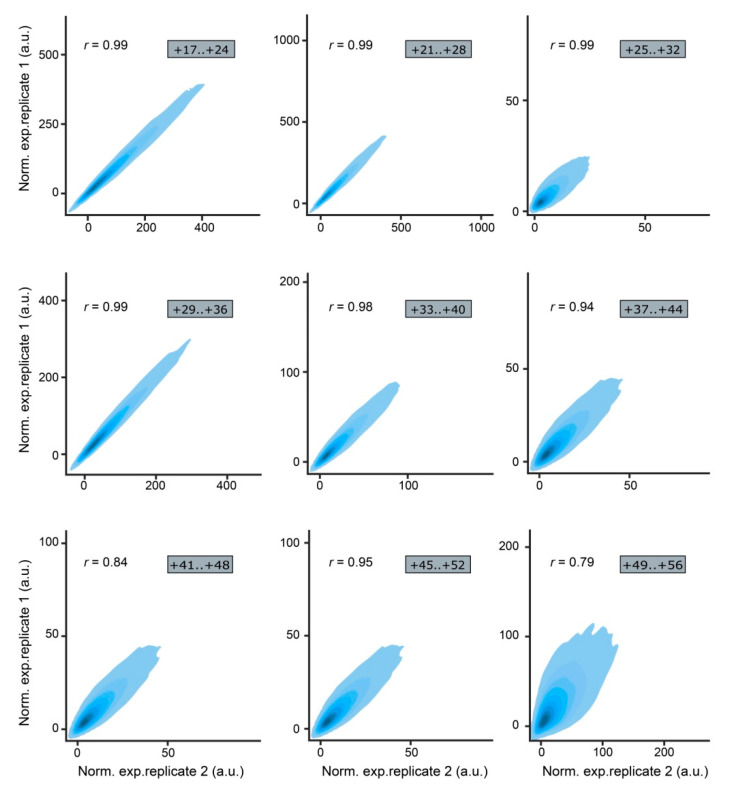
Correlation of normalized expression values between the replicates of the MPRA libraries visualized as density scatterplots; *r* denotes Pearson’s correlation coefficient.

Analysis of the NGS reads was performed using the previously described MPRAdecoder pipeline [93]. It revealed that the MPRA libraries have different numbers of unique mutations (Table 1). On average, 54%, 14%, and 9% of mutations across the libraries were associated with one, two, and three or more BCs, respectively. It should be noted that a high correlation of normalized expression values between the replicates was obtained for all MPRA libraries (Figure 3).

**Figure 4:**
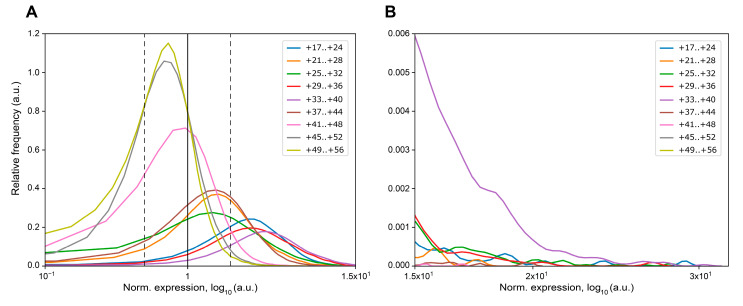
Kernel density estimation of normalized expression values for the MPRA libraries that were averaged over replicates and normalized to the wildtype reference construct. (**A**) Density plot with a scale ranging from 1.0 × 10^−1^ to 1.5 × 10^1^. The expression level of the wildtype reference construct is indicated by a solid black vertical line. The twofold changes in the expression levels are depicted by dotted black vertical lines. (**B**) Density plot with a scale ranging from 1.5 × 10^1^ to 3.2 × 10^1^.

Next, we evaluated the number of mutations in each MPRA library that increase or decrease *eGFP* expression more than twofold compared to the wildtype reference construct (Table 2, Figure 4). We found that more than 50% of mutations in ROIs +17..+24, +21..+28, +25..+32, +29..+36, and +33..+40 result in more than a twofold increase in *eGFP* mRNA. At the same time, a proportion of mutations decreasing *eGFP* expression more than twofold was very low (0.07–3.21%) in these ROIs, as well as in ROI +37..+44. Additionally, these ROIs were characterized by the presence of mutations increasing *eGFP* expression more than 20-fold, whereas, in the ROIs +41..+48, +45..+52, and +49..+56 about 80% of mutations did not increase the *eGFP* reporter gene expression (Table 2, Figure 4).

**Figure 5:**
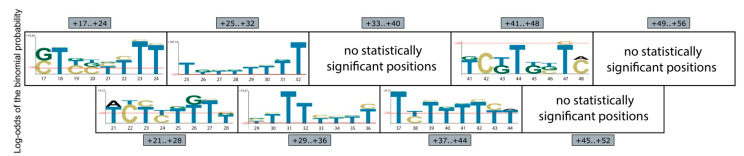
Position weight matrix logos for sequences that dramatically increase (≥10-fold) *eGFP* mRNA level according to the MPRA screening results. A significance p-value threshold level of 0.05 is indicated by red horizontal lines [94].

Then, we analyzed the nucleotide composition of mutations leading to more than a 10-fold increase in *eGFP* mRNA abundance according to the NGS data. With the exception of three ROIs, it was possible to define consensuses of sequences favorable for gene expression using the pLogo software [94]. These consensus sequences appeared to be extremely T-rich (Figure 5).

**Figure 6:**
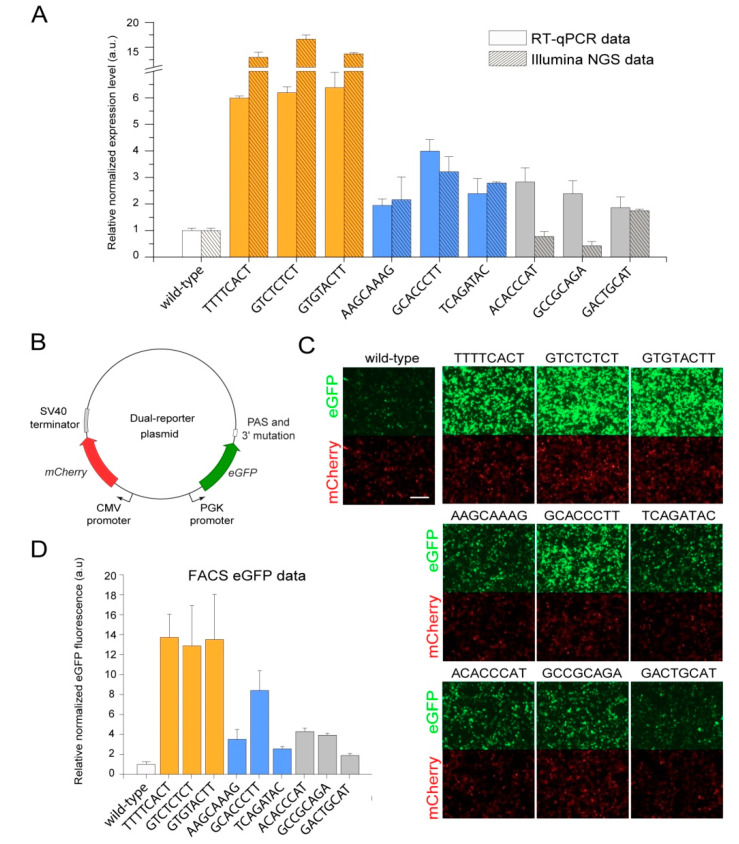
Validation of the positive effects of several chosen mutations identified in the MPRA screening on *eGFP* expression at the transcript and protein levels in HEK293T cells. (**A**) Comparison of *eGFP* expression data obtained for individual mutations using RT-qPCR with data obtained using Illumina NGS. Both types of data were normalized by the expression level of the wildtype reference construct. (**B**) The scheme of the dual-reporter plasmid used to test individual mutations. (**C**) Microscopy detection of *eGFP* and mCherry fluorescence intensities in HEK293T cells transiently transfected with the plasmids bearing the indicated individual mutations. Scale bar, 50 μm. (**D**) Flow cytometry analysis of the *eGFP* protein expression in HEK293T cells transiently transfected with the plasmids bearing the indicated individual mutations.

To verify the effects of individual mutations on *eGFP* expression level detected by the NGS analysis, we individually cloned several particular mutations from the library +29..+36 that change *eGFP* expression to a different extent downstream of a non-barcoded *eGFP* reporter. The mutations represent the “high”-, “medium”-, and “low”-expression groups. Each group included three mutations with different read counts in the normalization replicates (Table 3). HEK293T cells were transfected individually by non-barcoded plasmids bearing these mutations and by the non-barcoded wildtype reference construct. Then, 48 h after transfection, we analyzed the levels of *eGFP* mRNA and protein by RT-qPCR, fluorescence microscopy, and flow cytometry assays (Figure 6). Overall, the results of all approaches used were in good accordance with each other, although, on average, lower and higher fold changes were detected by RT-qPCR and flow cytometry assays compared to the NGS data for mutations from the high and medium/low groups, respectively (Figure 6).

**Figure 7:**
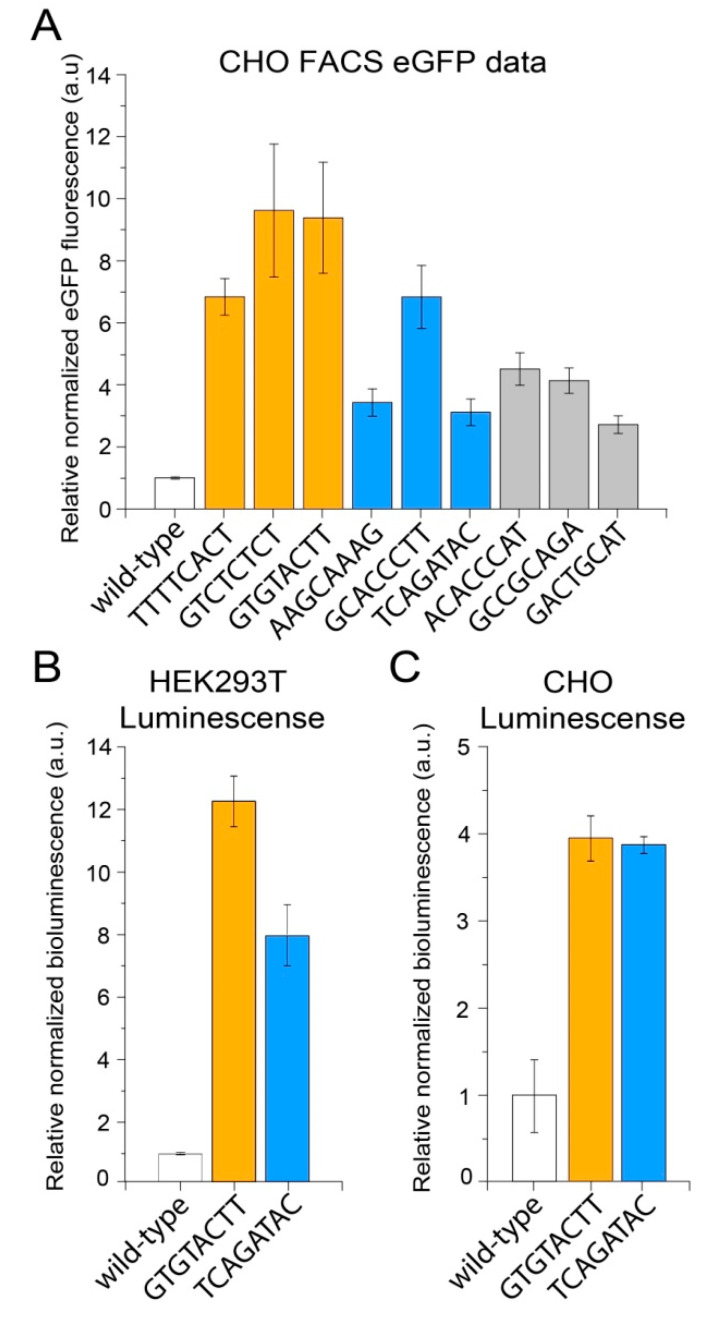
The positive effects of GTGTACTT and TCAGATAC mutations on the expression levels of different upstream reporter genes and in different cell lines. (**A**) Flow cytometry analysis of the *eGFP* protein expression in CHO cells transiently transfected with the plasmids bearing the indicated individual mutations. (**B**,**C**) The luciferase protein activity in HEK293T (**B**) and CHO (**C**) cells transiently transfected with the plasmids bearing the indicated individual mutations.

After that, we sought to determine whether the positive effects of mutations on *eGFP* expression could be reproduced in another mammalian cell line, as previously had been demonstrated for the deltaC mutation [89]. For that, we transiently transfected CHO cells using the same set of non-barcoded plasmids bearing individual mutations and analyzed the *eGFP* protein levels by flow cytometry (Figure 7A). This revealed a high level of conservation of the found positive effects of mutations on *eGFP* expression.

Lastly, we decided to check whether the effects are limited to the *eGFP* reporter used in all experiments described above or are a more general phenomenon. With this aim, the two mutations, GTGTACTT from the high-expression group and TCAGATAC from the medium-expression group, which were originally supported by the highest numbers of reads in the NGS data, as well as the wildtype sequence, were individually cloned downstream of a non-barcoded *luciferase* (*NanoLuc*) reporter. Flow cytometry analysis of HEK293T and CHO cells transfected by these plasmids revealed that both mutations also significantly increased the luciferase protein activity in both cell lines (Figure 7B,C). Interestingly, both mutations affected the luciferase protein expression to the same extent in CHO cells, suggesting cell-type-specific variations in the transcription termination process. Overall, these findings suggest that the MPRA assays can be effectively used to identify sequence variants in the 3′ end of genes that are likely not included in the mature transcripts but substantially increase gene expression levels.

## 3. Discussion

Molecular mechanisms of transcription termination in eukaryotes are actively studied. However, the extent to which and particular ways of how the DNA sequence in the 3′ region of a gene downstream the PAS can affect the number of mature transcripts (usually considered as a level of gene expression) remain intriguing questions. In the present study, we used an artificial *eGFP* reporter gene and the MPRA approach to elucidate the regulatory effects of mutations downstream of the PAS.

We found that the majority of mutations located distal to the PAS have no positive effect on *eGFP* expression (Figure 4, Table 2). At the same time, the majority of mutations in the ROIs located proximally to the PAS cause a significant increase in *eGFP* mRNA level (up to 30-fold compared to the wildtype reference construct) (Figure 4, Table 2). For mutations resulting in the decrease in *eGFP* expression in the ROIs proximal to the PAS, we found very low number of cases causing a twofold or greater impact (or having zero counts in the expression replicates). The proportion of such mutations in the distal ROIs increased up to 11–18% (Table 2).

To verify the NGS data, we also analyzed the effects of individual mutations on *eGFP* expression using the RT-qPCR assay. As a result, we observed a very high correlation between RT-qPCR and NGS data for mutations from high- and medium-expression groups (Pearson’s correlation coefficient *r* = 0.94). We did not observe any difference between mutations in these groups depending on the read counts in the normalization replicates (Figure 6A, Table 3). However, the correlation between data obtained using RT-qPCR and NGS for mutations from the low-expression group was very low (Pearson’s correlation coefficient *r* = −0.75). Despite that, the correlation among these data for mutations from all three groups was high (Pearson’s correlation coefficient *r* = 0.94) (Figure 6A). It should be noted that, 48 h after transfection, we also performed a microscopic analysis of *eGFP* fluorescence in HEK293T cells. The level of *eGFP* signal in cells correlated well with the NGS and RT-qPCR data (Figure 6C). Thus, we can conclude that the NGS data obtained for the mutations leading to the twofold and more increase in *eGFP* expression level are reliable. At the same time, the NGS data on mutations that just slightly change the *eGFP* expression should be interpreted with caution. We suppose that this observation does not detract from the merits of the present study, since, in most cases, the need is to increase the expression of the gene of interest not slightly but substantially.

We also found that the consensus of sequences that ensure the highest expression levels of the *eGFP* reporter is extremely T-rich (Figure 5). Moreover, statistically significant nucleotides were identified only in the ROIs located proximal to the PAS. In the distal ROIs, no statistically significant nucleotides leading to a high increase in *eGFP* mRNA expression were detected.

As the MPRA assays are based on the usage of BCs to tag sequences absent in mature transcripts, it is important to note that BCs can also affect the measurements by themselves [95]. Typically, this issue is solved by associating each mutant sequence with multiple BCs [91,96]. Although such a strategy was not implemented in the current study, we believe that the strong positive effects on the reporter gene expression were not primarily caused by the BCs. First, the correlation between normalized expression values obtained with different BCs associated with the same mutations was on average 2.79 times higher for mutations increasing *eGFP* expression ≥2-fold than for the entire pool of analyzed mutations. Second, we identified similar trends in nucleotide composition (which is an enrichment in Ts) for mutations from different ROIs that strongly increase *eGFP* expression level. This suggests that the sequences of these mutations are more important than the sequences of BCs associated with them. Third, for several mutations from the high- and medium-expression groups, we validated the MPRA NGS data by RT-qPCR, fluorescence microscopy, and flow cytometry approaches using non-barcoded plasmids. Thus, although the effects of BCs cannot be completely ruled out, their influence on the identification of mutations strongly increasing the *eGFP* reporter expression level seems to be very small.

We assume that the effects of an increase in the reporter expression due to mutations that we detected stemmed from an increase in the efficiency of assembly and/or function of the CPA complex. At the moment, we can only speculate on the exact mechanism of such an increase in the CPA complex efficiency, but further investigation of results presented in this study could aid in the elucidation of specific mechanisms. A large body of evidence suggests the importance of the 3′ gene region composition for the correct completion of transcription termination by the CPA complex action. Previously, a massive analysis of human genomic sequences surrounding frequently and less frequently used poly(A) sites revealed specific *cis* elements within human 3′ gene regions [32]. Recent transient transcriptome sequencing (TT-seq) studies showed that the termination window of human genes can reach several kbs, and this region is thought to contain motifs crucial for the termination of transcription. Termination windows were found to be highly enriched with the consensus motif (C/G)_(2–6)_AN_x_(T/A)_(3–6)_ (where N_x_ is a short stretch of nucleotides). The (C/G)_(2–6)_ motif was shown to be associated with the paused state of the RNAP II, and (T/A)_(3–6)_ may promote dissociation of the CPA complex due to the low melting temperature of the DNA–RNA hybrids [97,98]. It is also known that RNA forms secondary structures that are extremely important for RNA–protein interactions [99], such as the assembly of the CPA complex. We found a notable difference between predicted secondary structures of mRNA molecules bearing individual mutations from high- and low-expression groups (Supplementary Figure S1). As there are no exact data on which RNA secondary structures correlate with a more stable CPA complex, we hypothesize that, in the case of low-expression mutations, the specific secondary structure prevents the binding or interaction of individual subunits of the CPA complex.

Previously, we showed that deltaC, a single-nucleotide deletion in the 3′ region of the *eGFP* reporter affects the choice of the cleavage/polyadenylation sites of the transcripts. Thus, differences in (i) the composition of the 3′ UTR, (ii) the proportion of polyadenylated transcripts, and (iii) the length of the polyA tail can be expected in mRNAs synthetized from the mutated templates described in the present study. All these parameters affect the translation efficiency and lifetime of the resulting transcripts (and, therefore, their detectability) [72,73,100]. The whole-transcriptome 3′ UTR-seq of tens of thousands of transcript sequences in zebrafish revealed that stabilizing polyU and UUAG sequences and destabilizing GC-rich signals regulate early-onset rates of mRNA degradation [101].

We also cannot rule out the possibility of other related mechanisms being involved. In particular, the relationship between transcription termination and re-initiation is well established; hence, the efficiencies of these processes are interrelated [18,102]. The RNAP II dissociation from the 3′ end of a gene and the subsequent transcription machinery transition to the active state depend on the rate and success of transcription termination completion. Therefore, some effects observed in this study might also be explained by changes in the transcription re-initiation process. In this respect, it should be noted that a head-to-head arrangement of genes implemented in the dual-reporter plasmids used to verify the results of MPRA screening was reported to be associated with co-regulation of such genes due to simultaneous assembly and initiation of the transcription machinery on their transcription start sites [103]. However, our present and previous data [89] show that the *mCherry* normalization gene (Figure 6B) expression level is stable and does not depend on different motifs present in the 3′ region of the *eGFP* reporter. Therefore, we think that the effects of the 8 bp mutations on the upstream reporter gene expression are rather not strongly associated with the transcription re-initiation.

We believe that the results of the present work may be interesting for use in the production of therapeutic proteins and viral vectors [104,105,106], since HEK293T and CHO are the most popular cell lines for these purposes [107,108,109,110,111,112]. Since one of the important tasks in this field is to increase production efficiency, the modification of sequences involved in the transcription termination of transgenes based on the results of the appropriate MPRA screenings may substantially contribute to solving this problem.

## 4. Materials and Methods

### 4.1. Gibson Assembly of MPRA Plasmid Libraries

To generate the pTTC-hPGK-eGFP construct, the pTTC-Hsap-WT plasmid [89] was digested with NheI and XbaI and self-ligated. MPRA plasmid libraries were constructed on the basis of the pTTC-hPGK-eGFP plasmid using the Gibson Assembly approach. For each library, the appropriate DNA fragments were amplified from the pTTC-hPGK-eGFP plasmid template using primers indicated in Supplementary Tables S1 and S2. The PCR amplification was conducted in a 50 µL volume containing 1 ng of plasmid DNA, 1× Phusion high-fidelity reaction buffer (Thermo Fisher Scientific, Waltham, MA, USA), 400 µM of each dNTP, 500 nM of each primer, and 1 U of Phusion high-fidelity Hot Start DNA Polymerase (Thermo Fisher Scientific, Waltham, MA, USA) with the following program: 98 °C for 1 min, followed by 35 cycles of 98 °C for 30 s, 60 °C for 30 s, and 72 °C for 3 min, with a final cycle at 72 °C for 5 min. The PCR products were digested with DpnI restriction enzyme (New England Biolabs, Ipswich, MA, USA) at 37 °C for 4 h in a volume of 100 μL in order to destroy the plasmid template and, thus, minimize non-barcoded vector contamination in the libraries. After purification with the PCR Purification Kit (Qiagen, Hilden, Germany), 76 fmol (200 ng) of “vector” and 0.38 pmol (~30 ng) of “insert” fragments were incubated with Gibson Assembly Master Mix (New England Biolabs, Ipswich, MA, USA) at 50 °C for 1 h. Then, the reaction mixture was diluted 10-fold with nuclease-free water and purified using the MinElute PCR Purification Kit (Qiagen, Hilden, Germany); DNA was eluted by 10 µL of prewarmed elution buffer. Then, 5 μL of the purified Gibson cloning reaction was used to transform *E. coli* TOP10 electrocompetent cells.

### 4.2. Generation of Reference Barcoded Plasmid Constructs

To generate two reference constructs with the original “wildtype” sequence of the transcription terminator marked by specific 20 bp BCs, the pTTC-hPGK-eGFP plasmid was digested with XhoI and MluI. The barcoded wildtype sequence was amplified from the pTTC-hPGK-eGFP plasmid template using primers 5′–GACACTCGAGGATCGAGTTCCAAGTGCAGGTTAGGCGGAGTTGTGGCCGGCCCTTG–3′ (20 bp BC_1_) or 5′–GACACTCGAGGATCGAGTGTGTACGGCTTGCTCTCAAGAGTTGTGGCCGGCCCTTG–3′ (20 bp BC_2_) and 5′–CGCATACGCGTATACTAGATTAACC–3′. The PCR amplification was conducted in 50 µL volume containing 1 ng of plasmid DNA, 1× Phusion high-fidelity reaction buffer (Thermo Fisher Scientific, Waltham, MA, USA), 400 µM of each dNTP, 500 nM of each primer, and 1 U of Phusion high-fidelity Hot Start DNA Polymerase (Thermo Fisher Scientific, Waltham, MA, USA) with the following program: 98 °C for 1 min, followed by 35 cycles of 98 °C for 30 s, 57 °C for 30 s, and 72 °C for 30 s, with a final cycle at 72 °C for 5 min. The PCR products were digested with XhoI and MluI at 37 °C for 1 h. After purification with the PCR Purification Kit (Qiagen, Hilden, Germany), 50 ng of “vector” and ~7 ng of “insert” fragments were ligated at +4 °C overnight with T4 DNA ligase (Evrogen, Moscow, Russia). Then, 1 μL of the ligation mixture was used to transform *E. coli* TOP10 electrocompetent cells.

### 4.3. Generation of Non-Barcoded Plasmid Constructs with Individual Mutations

To generate the non-barcoded plasmid constructs with individual mutations, the pTTC-Hsap-WT plasmid [89] was first modified by introducing a point mutation in the SpeI site located within the human *PGK* promoter driving the expression of the *eGFP* reporter (cloning details are available upon request). Importantly, the introduced mutation did not affect the activity of the promoter. Next, the modified pTTC-Hsap-WT plasmid was digested at unique restriction sites with SpeI and BsiWI. For each mutation, two single-stranded complementary oligonucleotides (5′–CTAGTCATGCGTCAATTNNNNNNNNGATTATCTTTAAC–3′ and 5′–GTACGTTAAAGATAATCNNNNNNNNAATTGACGCATGA–3′) were annealed to each other by heating their equimolar mixture to 94 °C and then gradually cooling it to room temperature, which resulted in double-stranded DNA fragments with the SpeI and BsiWI sticky ends. After purification of digested vector with the PCR Purification Kit (Qiagen, Hilden, Germany), 50 ng of “vector” and ~1 ng of “insert” fragments were ligated at +4 °C overnight with T4 DNA ligase (Evrogen, Moscow, Russia). Then, 1 μL of the ligation mixture was used to transform *E. coli* TOP10 electrocompetent cells.

To generate the non-barcoded plasmid constructs with individual mutations and the *luciferase (NanoLuc)* reporter, the plasmid constructs with wildtype sequence or GTGTACTT and TCAGATAC mutations were digested with SmaI and EcoRI. The *NanoLuc* coding sequence was amplified from the plasmid template (provided by the laboratory of antibody engineering, IMCB SB RAS) using primers 5′–ATAACCCGGGACCATGGTCTTCACACTCG–3′ and 5′–ATATGAATTCTTACGCCAGAATGCGTTCG–3′. The PCR amplification was conducted in 50 µL volume containing 1 ng of plasmid DNA, 1× Phusion high-fidelity reaction buffer (Thermo Fisher Scientific, Waltham, MA, USA), 400 µM of each dNTP, 500 nM of each primer, and 1 U of Phusion high-fidelity Hot Start DNA Polymerase (Thermo Fisher Scientific, Waltham, MA, USA) with the following program: 98 °C for 1 min, followed by 35 cycles of 98 °C for 30 s, 58 °C for 30 s, and 72 °C for 30 s, with a final cycle at 72 °C for 5 min. The PCR products were digested with SmaI and EcoRI at 37 °C for 1 h. After purification with the PCR Purification Kit (Qiagen, Hilden, Germany), 50 ng of “vector” and ~18 ng of “insert” fragments were ligated at +4 °C overnight with T4 DNA ligase (Evrogen, Moscow, Russia). Then, 1 μL of the ligation mixture was used to transform *E. coli* TOP10 electrocompetent cells.

All plasmid constructs were verified by Sanger sequencing.

### 4.4. Cell Lines and Transfections

Human embryonic kidney (HEK293T) cells were obtained from ATCC (USA). The HEK239T cells were cultured at 37 °C in Dulbecco’s modification of Eagle’s medium (DMEM; Gibco) with 10% fetal bovine serum (FBS; Gibco), 7.5% NaHCO_3_, 100 IU/mL of penicillin, and 100 μg/mL of streptomycin.

Chinese hamster ovary (CHO) cells were obtained from the laboratory of antibody engineering, IMCB SB RAS. The CHO cells were cultured at 37 °C in Iscove’s modified Dulbecco’s medium (IMDM; Gibco) with 10% fetal bovine serum (FBS; Gibco), 100 IU/mL of penicillin and 100 μg/mL of streptomycin.

Transient transfection of HEK293T and CHO cells was performed as described previously [89].

### 4.5. MPRA Sample Preparation for NGS

The MPRA samples were prepared as described previously [92]. Briefly, the mapping samples were prepared by two-round conventional PCR. The expression samples were prepared by PCR amplification of the BC sequences using 1/20 of cDNA as a template. The normalization samples were prepared by PCR amplification using plasmid libraries as templates. To accurately measure the concentration of the MPRA samples for Illumina NGS, we used quantitative real-time PCR as described previously [92].

### 4.6. Illumina NGS and Data Analysis

Sequencing of 151 nt-long single-end reads was performed on an Illumina MiSeq platform using the MiSeq Reagent Kit v3 150 cycles (Illumina). Fastq files were processed, and the data were analyzed using previously described MPRAdecoder script [93]. Position weight matrix logos for sequences that dramatically increase (≥10-fold) *eGFP* mRNA level according to the MPRA screening were defined using the pLogo software [94].

### 4.7. RNA Isolation and Quantitative Real-Time PCR

RNA isolation from cells was performed 48 h after transfection according to the previously described protocol [89]. The cells were lysed in 1 mL of TRIzol Reagent Solution (Thermo Fisher Scientific, Waltham, MA, USA), and total RNA was isolated according to the manufacturer’s instructions. Then, 10 μg of purified RNA was incubated with 3 U of DNase I (Thermo Fisher Scientific, Waltham, MA, USA) and 40 U of DpnI restriction enzyme (New England Biolabs, Ipswich, MA, USA) at 37 °C for 1 h to remove traces of genomic and plasmid DNA. The RNA was purified using the CleanRNA Standard kit (Evrogen, Moscow, Russia) according to the manufacturer’s instructions. Next, 1 μg of purified total RNA was reverse-transcribed into cDNA using an oligo(dT)_20_ primer and 100 U of RevertAid Reverse Transcriptase (Thermo Fisher Scientific, Waltham, MA, USA) according to the manufacturer’s instructions.

qPCR was performed using the BioMaster HS-qPCR SYBR Blue (2×) reagent kit (Biolabmix), CFX96 Real-Time PCR Detection System (Bio-Rad), and the following gene-specific primer pairs: 5′–CCCTTCCTGGCCATCCTG–3′ and 5′–TCATCTCATTGACTTTGTCCAGC–3′ for the human *PGK1* gene; 5′–CTTCAAGATCCGCCACAACATC–3′ and 5′–GGACCATGTGATCGCGCTTCTC–3′ for the *eGFP* coding sequence; 5′–ATCAAGGAGTTCATGCGCTTCAAG–3′ and 5′–TCACCTTCAGCTTGGCGGTCT–3′ for the *mCherry* coding sequence. The thermal cycling conditions were as follows: 5 min at 95 °C, followed by 39 cycles of 15 s at 95 °C, 30 s at 60 °C, and 30 s at 72 °C. No template control and no reverse transcriptase control samples were included in each run. Measurements of gene transcription levels were performed in two independent experiments, each with three technical replicates. The ΔΔCq method was used to calculate relative mRNA abundance; *eGFP* transcription levels were normalized to those of the *mCherry* and human *PGK1* genes.

### 4.8. Fluorescence Microscopy

Transfected cells were analyzed immediately before harvesting for RNA isolation. All samples were imaged at the same settings using an Axio Vert.A1 microscope (Zeiss, Jena, Germany) equipped with an AxioCam ICm1 camera (Zeiss, Jena, Germany).

### 4.9. FACS Analysis

Cells transfected as described above were harvested for FACS analysis 48 h after transfection. To calculate the level of the *eGFP* expression, the mean fluorescent intensity of *eGFP* positive cells at 510 nm was multiplied by the number of positive cells and divided by the total number of events in live cells using a FACSCanto II flow cytometer (Becton Dickinson).

### 4.10. Luciferase Reporter Assay

To measure NanoLuc activity, HEK293T and CHO cells transfected as described above were harvested for a luciferase reporter assay 48 h after transfection. Then, 800 μL of lysis buffer (PBS, 0.5% Triton X-100) was added to each well of a 12-well plate and incubated at room temperature for 15 min. Next, 5 μL of cell lysate was mixed with 95 μL of lysis buffer and 50 μL of coelenterazine h solution (NanoLight Technology, Pinetop, AZ, USA) in wells of a 96-well white plate (SPL Life Sciences, Pocheon-Si, South Korea). The bioluminescence signal of the sample was measured using a Luminoskan Microplate Luminometer (Thermo Fisher Scientific, Waltham, MA, USA).

## Figures and Tables

**Table 1:** Numbers of unique mutations and associated BCs for each MPRA library.

MPRA Library ID	Number of Unique Mutations	Mutations Associated with 1 BC		Mutations Associated with 2 BCs		Mutations Associated with ≥3 BCs	
		Number	%	Number	%	Number	%
+17..+24	8380	7567	90.3	731	8.7	82	1.0
+21..+28	8266	7347	88.9	797	9.6	122	1.5
+25..+32	39,705	21,267	53.6	11,018	27.7	7420	18.7
+29..+36	10,084	8830	87.5	944	9.4	310	3.1
+33..+40	27,232	19,346	71.0	5807	21.3	2079	7.7
+37..+44	33,866	19,721	58.2	8544	25.2	5601	16.6
+41..+48	56,470	20,208	35.8	15,077	26.7	21,185	37.5
+45..+52	25,814	18,373	71.2	5501	21.3	1940	7.5
+49..+56	17,938	11,888	66.3	3489	19.5	2561	14.2

**Table 2:** Number of mutations influencing the *eGFP* expression in the MPRA libraries.

MPRA Library ID	Number of Unique Mutations	Number of Mutations with Zero Counts in Expression Samples	Max Increase of *eGFP* Expression *	Max Decrease of *eGFP* Expression *	Mutations Leading to the ≥2-fold Increase of *eGFP* mRNA *		Mutations Leading to the ≥2-fold Decrease of *eGFP* mRNA *	
					Number	%	Number	%
+17..+24	8380	5	28.15	0.19	6961	83.07	22	0.26
+21..+28	8266	6	17.33	0.14	4474	54.13	79	0.96
+25..+32	39,705	160	27.38	0.05	23,204	58.44	1276	3.21
+29..+36	10,084	0	26.09	0.24	8724	86.51	7	0.07
+33..+40	27,232	11	29.45	0.23	25,323	92.99	25	0.09
+37..+44	33,866	58	19.67	0.07	16,304	48.14	746	2.20
+41..+48	56,470	277	16.74	0.01	6216	11.01	6187	10.96
+45..+52	25,814	25	7.59	0.01	1036	4.01	3771	14.61
+49..+56	17,938	249	6.74	0.02	518	2.89	3196	17.82

* Relative to the wildtype reference construct.

**Table 3:** Mutations from the MPRA library +29..+36 selected for individual tests.

Mutation Sequence	Normalization Replicate 1, Read Count	Normalization Replicate 2, Read Count	Increase of *eGFP* Expression, Fold *	Group
TTTTCACT	3	15	12.99	High expression
GTCTCTCT	11	11	16.63	
GTGTACTT	450	382	13.68	
AAGCAAAG	3	4	2.16	Medium expression
GCACCCTT	9	11	3.21	
TCAGATAC	281	264	2.78	
ACACCCAT	3	10	0.77	Low expression
GCCGCAGA	11	15	0.43	
GACTGCAT	92	64	1.75	

* Relative to the wildtype reference construct.

## Data Availability

Data supporting reported results can be downloaded from GEO (accession number GSE215681).

## Supplementary Materials

The following supporting information can be downloaded at https://www.mdpi.com/article/10.3390/ijms232415485/s1. References [113,114] are cited in the supplementary materials.
