# Supplementary material for: Slight Variations in the Sequence Downstream of the Polyadenylation Signal Significantly Increase Transgene Expression in HEK293T and CHO Cells
Source: Int J Mol Sci. 2022 Dec 7;23(24):15485. doi: 10.3390/ijms232415485 (PMC9779314; doi:10.3390/ijms232415485)
Supplement: Supplementary file 1 [file ijms-23-15485-s001.zip › ijms-2000471-supplementary.pdf]

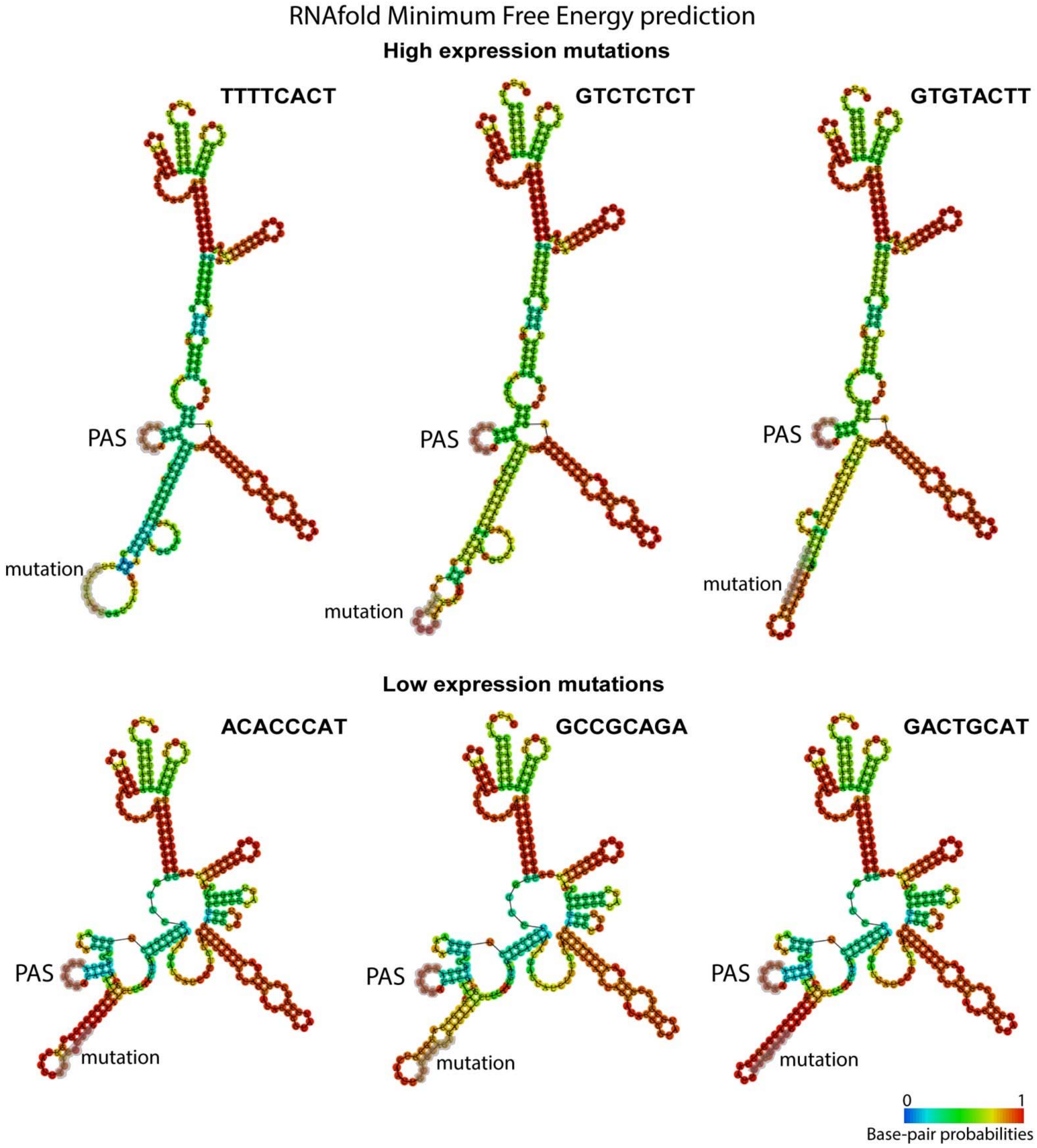


Figure S1. Prediction of the optimal secondary structure of the 3' region of *eGFP* mRNA for three "high" and three "low" expression mutations based on a minimum free energy of -75.70 kcal/mol using the RNAfold web server (http://rna.tbi.univie.ac.at/) [113,114]. The mRNA region used for the calculations spans from -75 nt to +200 nt relative to the PAS. The colour scale shows base-pair probabilities from 0 to 1. PAS – polyadenylation signal position; mutation – region of mutation. The regions of PAS and mutations are shown in grey.
